# Interconnections between the immune system and infertility in women with polycystic ovary syndrome: a scoping review

**DOI:** 10.61622/rbgo/2026rbgo39

**Published:** 2026-10-20

**Authors:** Lorena Sampaio da Silva dos Santos, Gabriel Reis Rocha, Gabriel Lima Correa Santos, Luís Guilherme de Oliveira Silva, Mariana Santos Calmon, Fabrício Freire de Melo

**Affiliations:** 1 Universidade Federal da Bahia Vitória da Conquista BA Brazil Universidade Federal da Bahia, Vitória da Conquista, BA, Brazil.

**Keywords:** Cytokines, Inflammation, Infertility, Polycystic ovary syndrome, Immune system

## Abstract

**Objective:**

To map the existing evidence on the influence of the immune system on infertility in women with polycystic ovary syndrome (PCOS).

**Methods:**

This scoping review was conducted in accordance with the Joanna Briggs Institute methodology and reported following the PRISMA-ScR guidelines. Searches were were conducted in January 2025 in PubMed, Embase, Virtual Health Library, LILACS, Web of Science, and Scopus for studies published between 2010 and 2024 in English, Portuguese, and Spanish. Study selection and data extraction were conducted independently by two reviewers. Data were analyzed descriptively and synthesized narratively, supported by charts and figures. This review considered primary studies published between 2010 and 2024 in English, Portuguese, or Spanish involving women of reproductive age with polycystic ovary syndrome, addressing the interplay between immune dysfunction and infertility. Gray literature, duplicate records, animal or in vitro studies, reviews, meta-analyses, and articles not aligned with the review objective were excluded.

**Results:**

A total of 19 studies were included, most of them observational and conducted predominantly in China and Middle Eastern countries. The main markers investigated were inflammatory cytokines, immune cells, and proteins related to oxidative stress. Increased levels of pro-inflammatory cytokines were observed, along with reduced levels of regulatory cytokines, consistently associated with anovulation and reproductive dysfunction. The evidence also demonstrated interactions between chronic low-grade inflammation, hormonal imbalance, and metabolic disturbances, which collectively contribute to impaired ovulatory function and reduced fertility.

**Conclusion:**

Infertility in PCOS is driven by a complex interplay between immune imbalance and endocrine–metabolic dysfunction, with chronic inflammation playing a central role in ovulatory and endometrial impairment. Despite these advances, the predominance of observational evidence and regional concentration of studies restrict broader applicability. These results underscore the need for well-designed research integrating immunological and reproductive pathways to inform more precise therapeutic strategies.

## Introduction

Polycystic Ovary Syndrome (PCOS) is an endocrinopathy of not yet fully elucidated etiology, prevalent among women of reproductive age. Its pathogenesis is associated with impaired feedback regulation of the hypothalamic–pituitary–ovarian (HPO) axis due to a disruption in the gonadotropin secretion pattern, resulting in luteinizing hormone (LH) hypersecretion, follicle-stimulating hormone (FSH) hyposecretion, and consequently, hyperandrogenism.^(1)^

The diagnosis is based on the Rotterdam Criteria (2003), requiring the presence of two out of the following three criteria: (i) amenorrhea or oligomenorrhea; (ii) clinical and/or biochemical hyperandrogenism; and (iii) polycystic ovarian morphology on ultrasonography, defined by the presence of 12 or more follicles measuring between 2 and 9 mm in diameter, or an ovarian volume greater than 10 cm³.^(2)^ Due to its multifactorial nature, PCOS is also recognized as a metabolic syndrome, characterized by the presence of hyperandrogenism, chronic anovulation, insulin resistance (IR) and obesity. These pathophysiological elements interact in an integrated manner, establishing it as one of the main causes of female fertility impairment.^(1,3)^

Infertility, in turn, is defined as the absence of conception after 12 months of regular sexual activity without the use of contraceptive methods in women under 35 years of age.^(4)^ In the context of PCOS, infertility may be triggered by multiple factors, environmental, genetic, and lifestyle-related, with anovulation being the main cause. During this process, ovarian tissue may be affected by immune responses resulting from chronic inflammation, such as the action of adipokines.^(5,6)^

Even in the absence of associated comorbidities, women with this endocrinopathy remain vulnerable to oxidative stress resulting from an imbalance between pro- and anti-inflammatory cytokines. This process promotes the establishment of a chronic low-grade inflammatory state in the ovarian endothelium, driven by immune system alterations that can impair ovulation.^(7)^ Such an immune response, combined with metabolic disturbances such as IR and hyperandrogenism, plays a central role in the syndrome's pathophysiology. These mechanisms, in addition to favoring the development of cardiovascular diseases, neoplasms, autoimmune conditions, and lipid metabolism disorders, are directly implicated in reproductive function.^(2)^

Therefore, PCOS is one of the most common causes of infertility in women of reproductive age, affecting 5% to 21% of patients and accounting for approximately 80% of anovulatory infertility cases.^(1,5)^ Among the pathophysiological mechanisms involved, immune imbalance stands out, promoting persistent inflammation in the ovarian microenvironment and potentially interfering with ovulation and embryonic implantation processes. However, the scarcity of data in the literature still poses significant challenges to understanding and managing the reproductive aspects of these patients.^(2)^

In this context, it is scientifically justified to expand knowledge on the interconnection between immunological dysfunction and infertility in women with PCOS, as in Brazil. Despite advances in reproductive medicine, the immunological causes related to the topic are still poorly explored. Current knowledge is fragmented, and there is a scarcity of studies that comprehensively integrate immunoinflammatory processes, which highlights a relevant gap in the literature.

From a practical perspective, addressing this knowledge gap has direct implications for the clinical management of infertility. Investigating this relationship may support the development of more individualized and effective therapeutic approaches, improving treatment protocols and promoting better reproductive outcomes and quality of life for patients.

The present scoping review aims to map the available evidence on the influence of the immune system on infertility in women with PCOS. The review was guided by the following research question: a) How does the interaction between the immune system and the pathophysiological mechanisms of PCOS influence the occurrence of infertility in affected women?; b) How are PCOS and chronic inflammation directly related to infertility outcomes?; c) In what way does the immunological status interfere with chronic anovulation?; d) How could immunological alterations be managed in the treatment of PCOS?. The research questions were structured according to the Population, Concept, and Context (PCC) model. The population (P) comprised women of reproductive age diagnosed with PCOS; the concept (C) referred to the immune system; and the context (C) corresponded to infertility.

## Methods

The review was conducted in accordance with guidelines outlined by the Joanna Briggs Institute (JBI) for scoping reviews, and followed the guidelines and checklist of the Preferred Reporting Items for Systematic reviews and Meta-Analyses extension for Scoping Reviews (PRISMA-ScR).^(8,9)^ The protocol of this review was registered and is publicly available on the Open Science Framework (OSF) platform (registration DOI: https://doi.org/10.17605/OSF.IO/68WHN).

### Eligibility criteria

Eligibility criteria were defined according to the PCC framework recommended for scoping reviews. Primary studies published between 2010 and 2024, in Portuguese, English, or Spanish, that addressed women with PCOS of reproductive age (19 to 35 years), infertility (anovulatory or unexplained), clinical and/or laboratory hyperandrogenism, oligomenorrhea and/or amenorrhea, and ultrasonographic alterations (≥12 follicles measuring 2–9 mm or ovarian volume >10 cm³), consistent with the Rotterdam criteria. The selected time frame was defined to capture contemporary evidence reflecting advances in immunological research in PCOS, and the age range defined to focus on women within the reproductive period, minimizing the confounding influence of physiological ovarian aging and age-related decline in fertility potential. The excluded studies were duplicated articles, gray literature (theses and dissertations), publications focused exclusively on the genetic factors of PCOS, in vitro or animal studies, meta-analyses, systematic reviews, and papers that did not directly address the proposed topic.

### Search strategy

To identify potentially relevant documents, the following bibliographic databases were searched from January 2010 to December 2024: PubMed, Embase (Excerpta Medica Database), Virtual Health Library (VHL), LILACS, Web of Science, and Scopus databases. No additional manual searches of reference lists, gray literature databases, or contact with study authors were performed.

Controlled vocabulary and free-text terms were combined using Boolean operators (AND/OR), with syntax adapted to each database (Chart 1S – Supplementary Material). The search strategy included the following combination of terms: *"Polycystic Ovary Syndrome" AND ("Infertility" OR "Anovulation" OR "Amenorrhea") AND ("Immune System" OR "Immune System Phenomena" OR "Inflammation" OR "Cytokines").*

In PubMed, phrase searching with quotation marks was performed without manual specification of field tags (e.g., [MeSH Terms] or [Title/Abstract]). This allowed Automatic Term Mapping (ATM) to identify corresponding Medical Subject Headings (MeSH) and related indexed terms according to the database structure. In Embase, searches were conducted using database-native syntax, allowing automatic mapping to Emtree terms when applicable. In VHL and LILACS, controlled descriptors indexed in DeCS and corresponding text words were applied according to the platform indexing system. In Web of Science, searches were performed across topic-related fields (title, abstract, and keywords). In Scopus, the search was restricted to title, abstract, and keyword fields (TITLE-ABS-KEY). These procedures ensured alignment with each database's indexing framework and enhanced the reproducibility of the search strategy.

### Selection process

All identified records retrieved from the database searches were collated and imported into Mendeley Desktop for duplicate removal. After this process, the references were uploaded to the Rayyan web-based platform to facilitate screening and study selection.

A standardized screening form was developed by the authors based on the predefined eligibility criteria and structured according to the Population–Concept–Context (PCC) framework. The form included guiding criteria addressing population characteristics (women of reproductive age diagnosed with PCOS), infertility-related outcomes, and the evaluation of immunological or inflammatory markers relevant to the review objective.

Before initiating the screening process, the reviewers discussed and agreed upon the interpretation of the eligibility criteria to ensure consistency in study selection. A formal pilot or calibration exercise of the screening form was not conducted. However, any uncertainties arising during screening were resolved through discussion to maintain alignment with the review objectives.

Study selection was conducted in two stages. In the first stage, two independent reviewers screened titles and abstracts according to the predefined inclusion and exclusion criteria. In the second stage, full-text articles of potentially eligible studies were retrieved and independently assessed for final inclusion. Reasons for exclusion at the full-text stage were documented. Any disagreements at any stage of the selection process were resolved through discussion and consensus. When necessary, a third reviewer was consulted to reach a final decision. The study selection process was documented and presented in a PRISMA-ScR flow diagram.

### Data extraction

Data extraction was performed independently by two reviewers using a standardized data extraction form developed by the authors in Microsoft Excel, based on the review questions and the PCC framework. The extraction tool was designed to systematically capture relevant information from each included study and was refined during the data extraction process to ensure completeness and consistency of the collected data. The data charting form was not formally piloted prior to implementation. Any modifications made to the extraction form during this process were documented in the review.

The extracted data included year of publication, country of origin, study design, diagnostic criteria for PCOS (Rotterdam, NIH, or AE-PCOS), infertility criteria, sample characteristics, evaluated immunological markers, hormonal and inflammatory parameters related to immune modulation, infertility-related outcomes, and reported study limitations. These elements were selected to directly address the review objectives and research questions.

All outcomes compatible with the predefined domains were extracted, regardless of the measurement method or timing. When multiple similar outcomes were reported, priority was given to baseline measurements and outcomes directly related to infertility.

### Quality analysis

A formal critical appraisal of methodological quality was not performed. This decision is consistent with the methodological purpose of scoping reviews, which aim to map and categorize available evidence rather than assess risk of bias, in accordance with JBI recommendations.

The analysis was conducted in a descriptive and narrative manner to address the study objectives and guiding questions, without calculating effect measures or performing quantitative comparisons. The results were organized into characterization charts and thematic syntheses, which allowed the categorization of findings into four main domains, immunological, inflammatory, hormonal, and metabolic, and their relationship with infertility, highlighting the frequency and nature of the markers associated with reproductive dysfunction in PCOS.

## Results

### Selection of sources of evidence

In the identification stage, a total of 2,128 articles were retrieved. After the removal of 1,255 duplicate records, 873 unique records remained and were screened by title and abstract, independently reviewed by two reviewers. Disagreements occurred in 147 records, which were subsequently assessed by a third reviewer to ensure consensus and consistency in the selection process. Following title and abstract evaluation, 812 records were excluded for not meeting the predefined eligibility criteria, and 61 articles were considered potentially relevant and therefore advanced to full-text assessment. At the eligibility stage, the full texts of these 61 articles were retrieved and carefully evaluated. Of these, 42 studies were excluded, 17 due to unavailability of the full text and 25 because they did not meet the established eligibility criteria upon detailed assessment. After completion of all screening phases, 19 studies fulfilled all inclusion criteria and were included in the final qualitative synthesis of this scoping review. The complete study selection process is presented in figure 1, in accordance with the PRISMA-ScR flow diagram.

**Figure 1:**
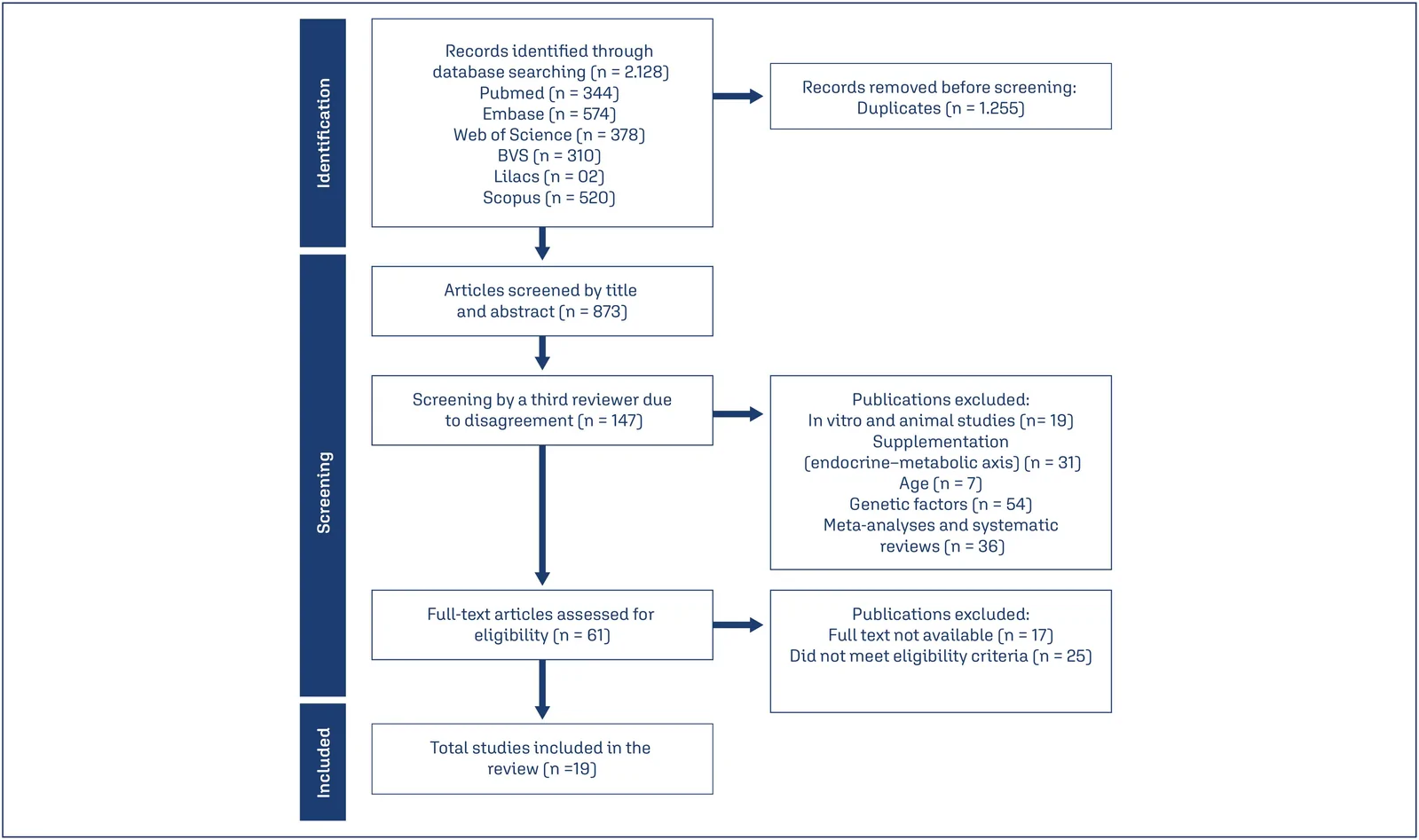
Study selection process.

### Characteristics of sources evidence

The included studies, published between 2010 and 2024, were conducted in eight different countries, as presented in chart 1. Most of the articles were concentrated in China, followed by Iraq and Iran.^(10-21)^

**Chart 1:** Characteristics of the included studies

Author(s)	Year	Title	Country	Study design	Number of participants	Age [Mean ± SD]	Associated health condition	Cause of infertility
Zhang et al.^(10)^	2017	Detection of dendritic cells and related cytokines in follicular fluid of patients with polycystic ovary syndrome	China	Case-control study	30	30.5 ± 1.2 years	Overweight; hyperandrogenism	Anovulation; poor oocyte or embryo quality; ovarian function marker (alteration in the follicular microenvironmen)
Zhou et al.^(11)^	2017	Increased expression of PGRN protein in follicular fluid and mRNA in granulosa cells in overweight patients with polycystic ovary syndrome	China	Comparative cross-sectional study	39	Group A (normal BMI): 29.7 ± 3.5 years Group B (obese): 31.2 ± 3.8 years	Obesity; Insulin resistance	Anovulation; Oocyte or embryo quality
Zhang et al.^(12)^	2020	IL-18 and IL-18 binding protein concentration in ovarian follicular fluid of women with unexplained infertility to PCOS during in vitro fertilization	China	Case-control study	60	29.47 ± 3.86 years	Obesity; Insulin resistance; Hyperandrogenism	Anovulation
Zhou et al.^(13)^	2022	The alterations of circulating mucosal-associated invariant T cells in polycystic ovary syndrome	China	Case-control study	33	29,45 ± 5,25 years	Obesity; Insulin resistance; Hyperandrogenism; Hyperinsulinemia	Anovulation
Liu et al.^(14)^	2021	The release of peripheral immune inflammatory cytokines promote an inflammatory cascade in PCOS patients via altering the follicular microenvironment	China	Case-control study	27	27.19 ± 3.32 years	Obesity; Insulin resistance	Anovulation; Oocyte or embryo quality
He et al.^(15)^	2020	Peripheral Blood Inflammatory-Immune Cells as a Predictor of Infertility in Women with Polycystic Ovary Syndrome	China	Case-control study	175	29 ± 4 years	Obesity; Insulin resistance	Anovulation
Al-Shattawi e Al-Jumili^(16)^	2019	The effect of hypercytokinemia in the pathogenesis of polycystic ovary syndrome in Iraqi women	Iraq	Cross-sectional study	50	26.38 ± 0.63 years	Obesity; Insulin resistance; Thyroid disorder	Anovulation
Al-Kubaisi e Al-Abassi^(17)^	2019	Determination of IL-18, C3 and insulin in Iraqi females with polycystic ovary syndrome (PCOS)	Iraq	Case-control study	66	28.09 ± 0.59 years	Obesity; Insulin resistance; Hyperandrogenism	Anovulation
Al-Janabi^(18)^	2019	Comparative study of hormonal and IL-6 levels among infertile women with polycystic ovary syndrome and healthy women	Iraq	Comparative cross-sectional study	90	28 ± 4.7 years	No data reported	Anovulation
Nasri et al.^(19)^	2018	T helper cells profile and CD4+CD25+Foxp3+ regulatory T cells in polycystic ovary syndrome	Iran	Case-control study	10	27 ± 1.2 years	Insulin resistance; Overweight	Anovulation
Sadeghi et al.^(20)^	2020	Circulating levels of C1q/TNF‐α‐related protein 6 (CTRP6) in polycystic ovary syndrome	Iran	Case-control study	120	31.0 years	Obesity; Insulin resistance	Anovulation
Shirvanizadeh et al.^(21)^	2023	Abdominal obesity may play a significant role in inflammation exacerbation in polycystic ovary syndrome patients	Iran	Observational cross-sectional study	20	Group A (normal BMI): 28.25 ± 2.25 years Group B (obese): 28.8 ± 3.32 years	Obesity; Hyperandrogenism	Anovulation; Oocyte or embryo quality
Younis et al.^(22)^	2014	Serum tumor necrosis factor-α, interleukin-6, monocyte chemotactic protein-1 and paraoxonase-1 profiles in women with endometriosis, PCOS, or unexplained infertility	USA	Prospective observational study	36	30.5 ± 4.8 years	Obesity; insulin resistance	Anovulation; impaired endometrial receptivity; endometrial abnormality
Paravati et al.^(23)^	2020	Differential regulation of osteopontin and CD44 correlates with infertility status in PCOS patients	Pakistan	Case-control study	43	29.1 ± 2.55 years	Obesity; Hyperandrogenism	Anovulation; Altered endometrial receptivity
Li et al.^(24)^	2021	Increased testosterone and proinflammatory cytokines in patients with polycystic ovary syndrome correlate with elevated GnRH receptor autoantibody activity assessed by a fluorescence resonance energy transfer-based bioassay	USA	Case-control study	33	29.9 ± 2.1 years	Obesity; Insulin resistance	Anovulation
Koc et al.^(25)^	2017	Nuclear factor-jB expression in the endometrium of normal and overweight women with polycystic ovary syndrome	Turkey	Case-control study	35	Group A (normal BMI): 28.05 ± 3.7 years Group B (obese): 28.1 ± 4.9 years	Obesity; Insulin resistance; Hyperandrogenism	Anovulation
Shruthi et al.^(26)^	2024	Increased circulating levels of novel anti-inflammatory cytokines IL-27 and IL-38 are associated with immunoendocrine dysregulation and altered redox stress in polycystic ovarian syndrome	India	Observational cross-sectional study	45	Under 50 years (Mean ± SD not specified)	Obesity; Insulin resistance; Hyperandrogenism	Anovulation
Sylus et al.^(27)^	2020	Matrix metalloproteinase-9 increases and interleukin-10 reduces with increase in body mass index in polycystic ovary syndrome: A cross-sectional study	India	Cross-sectional study	62	24.7 ± 2.3 to 26.1 ± 3.5 years	Obesity	Anovulation
Matteo et al.^(28)^	2010	Reduced percentage of natural killer cells associated with impaired cytokine network in the secretory endometrium of infertile women with polycystic ovary syndrome	Italy	Experimental clinical study	28	28 ± 8 years	Obesity	Anovulation; impaired endometrial receptivity; poor oocyte or embryo quality

The methodological designs of the studies varied, including prospective observational studies, case-control studies, cross-sectional studies, and experimental clinical studies.^(10,12-15,17,19-28)^

The study samples consisted of women of reproductive age diagnosed with PCOS, established according to the Rotterdam criteria, which were uniformly adopted across all selected studies. The number of participants per study varied, with the smallest including 10 infertile women and the largest comprising 175 women, totaling 1,002 participants. Analysis of the participants’ mean age revealed variations ranging from 24.7 ± 2.3 to 31.2 ± 3.8 years, with an overall mean of approximately 28.5 ± 3.5 years.

The health conditions associated with PCOS were reported at different frequencies across the analyzed studies (Chart 1). The most common conditions were obesity, IR, and hyperandrogenism.^(10-28)^ Other reported conditions included overweight, hyperinsulinemia^(13)^, and thyroid disorders.^(10,13,16,19)^ Only one study did not provide data on health conditions associated with infertility in women with PCOS.^(18)^

In the context of infertility, heterogeneity was also observed in the diagnostic criteria, as presented in Chart 1. Anovulation was the most frequently reported cause.^(10-28)^ Other described criteria included impaired oocyte or embryo quality, alterations in endometrial receptivity, as well as endometrial abnormalities and changes in ovarian function markers.^(10,11,14,21-23,25,28)^

## Synthesis of results

### Evaluation of immunological markers and their correlation with infertility

The analyzed studies demonstrate that infertility in PCOS is associated with multiple alterations of immunological, inflammatory, hormonal, and metabolic nature. From the data analysis, nine main categories of immunological markers investigated in the included studies were identified. For the purpose of standardizing the presentation of results, it is important to note that the values (n = x) mentioned throughout the text refer to the number of studies that evaluated each marker, as shown in chart 2.

**Chart 2:** Immunological and metabolic markers: Types, correlation with infertility, and frequency in the included studies

Markers	Types	Regulation in PCOS	Correlation with infertility	n(%)
Cytokines	TNF-α e IFN-γ^(10,11,13,14,17,18,21-24,26)^	↑	↓ Oocyte quality ↓ Embryo implantation ↓ Follicular maturation ↓ Ovulatory function ↓ Estradiol and other reproductive hormones	15(78.9)
	IL-17^(13)^	↑	↓ Embryo implantation and ovulation	
	IL-6^(10,11,16,21,22,24,26)^	↑	↓ Endometrial receptivity	
	MCP-1^(22)^	↑	Affects the follicular microenvironment and the mechanisms of follicular rupture	
	IL-1β^(26)^	↑	↓ FSH and LH receptors ↓ Estradiol ↑ Abnormal follicular cell proliferation ↓ Follicular maturation	
	IL-18^(12,21,28)^	↑/↓	↓ Embryo quality and the number of mature oocytes Implantation failure	
	IL-22^(13)^	↓	↓ Ovarian function	
	IL-23^(10)^	↓	↓ Follicular development	
	IL-10^(27)^	↓ (in obese women)	Low embryo implantation rate	
	TGF-β e IL-15^(26,28)^	↓	Affect endometrial immunomodulation ↓ Embryo implantation	
Immune system cells	CD4: Th1, Th17, Th1/Th2 ratio, and Th17/Treg ratio (pro-inflammatory profile)^(19,23)^	↑	↓ Endometrial receptivity and implantation	6 (31.6)
	Treg cells, MAIT cells, CD56^bright/CD16⁻ NK cells, DCs^(13,15,28)^	↓	↓ Immune regulation	
	T CD3⁺^(19)^	↑	Impaired embryo implantation	
	Lymphocytes CD11c⁺ HLA-DR⁺^(10)^	↓	Affects folliculogenesis and follicular selection ↓ Oocyte quality	
Adipokines	Adiponectin^(26)^	↑/↓	↓ Follicular/ovulatory function	3(15.8)
	Leptin^(26)^	↓		
	PGRN^(11)^	↑	↓ Oocyte number	
	CTRP6^(20)^	↑	↑ Insulin resistance Lipid dysfunction	
	FGF-21^(26)^	↑		
Oxidative stress	PON-1^(22)^	↑	↓ Folliculogenesis and embryogenesis ↓ Oocyte maturation ↓ Endometrial receptivity	3(15.8)
	Plasma nitrate/nitrite^(26)^	↑	↓ Oocyte maturation	
	ROS^(14)^	↑		
Autoantibodies	GnRHR-AAb^(24)^	↑	↓ Ovulatory function	1(5.3)
Chemokines	SDF-^1(26^)	↓	↓ Follicular function	2(10.5)
	CXCL10^(28)^	↓	Tissue remodeling and ↓ endometrial receptivity	
Metabolic markers	Insulin and Fasting blood glucose^(20,25)^	↑	Endometrial inflammation and ↓ embryo implantation	2(10.5)
	Testosterone (total and free)^(20,25)^	↑		
	HOMA-IR^(25)^	↑		
	HDL-C^(20,25)^	↓	Low-grade chronic inflammation Anovulation	
	TG e LDL-C^(20,25)^	↑		
	LH^(20,25)^	↑	Anovulation Failure of follicular maturation ↓ Oocyte quality and granulosa cell function	
	FSH^(20,25)^	↓		
Inflammatory proteins	NF-κB (p65 e p-p65) e NLRP3^(14,25)^	↑	↓ Oocyte quality	3(15.8)
	Homocysteine and hs-CR^P(20^)	↑	↑ Low-grade inflammatory state ↑ Risk of recurrent pregnancy loss	
Others	NO^(27)^	↓	↓ Endometrial/placental function	5(26.3)
	MMP-9^(27)^	↑	Ovarian/uterine remodeling	
	TSH, T3^(16)^	↑	Mild hypothyroidism: ↓ Implantation ↓ Endometrial function	
	T4^(16)^	↓		
	C3^(16)^	↑	Systemic inflammation	
	OPN–CD44 complex (endometrium / serum levels)^(23)^	↓ / ↑	↓ Endometrial receptivity	
	Granzyme B^(12)^	↑	↓ Oocyte maturation	

↑ increase; ↓ decrease; ↑/↓ increase or decrease.; TNF-α: tumor necrosis factor alpha; IFN-γ: interferon gamma; IL: interleukin; MCP-1: monocyte chemoattractant protein-1; FSH: follicle-stimulating hormone; LH: luteinizing hormone; TGF-β: transforming growth factor beta; CD: cluster of differentiation; Th1/Th2/Th17: T helper lymphocyte subsets; Treg: regulatory T lymphocytes; MAIT cells: mucosal-associated invariant T cells; NK cells: natural killer cells; CD56^bright/CD16^–^: immature NK cell subset; DCs: dendritic cells; CD3^+^ T cells: T lymphocytes identified by CD3 expression; CD11c^+^ HLA-DR^+^: antigen-presenting cell subpopulation associated with dendritic cells and activated monocytes; HLA-DR^+^: human leukocyte antigen – DR isotype positive; Adiponectin: peptide hormone secreted by adipose tissue with anti-inflammatory and insulin-sensitizing activity; Leptin: adipose-derived hormone that regulates satiety and energy metabolism; PGRN: progranulin; CTRP6: complement C1q tumor necrosis factor-related protein 6; FGF-21: fibroblast growth factor 21; PON-1: paraoxonase 1; ROS: reactive oxygen species; GnRHR-AAb: autoantibodies to the gonadotropin-releasing hormone receptor; SDF-1: stromal cell–derived factor 1; CXCL10: C-X-C motif chemokine ligand 10; HOMA-IR: Homeostasis Model Assessment of Insulin Resistance; HDL-C: high-density lipoprotein cholesterol; LDL-C: low-density lipoprotein cholesterol; TG: triglycerides; NF-κB (p65 and p-p65): nuclear factor kappa B (p65 subunit and phosphorylated p65); NLRP3: NOD-like receptor protein 3 (inflammasome); Homocysteine: sulfur-containing amino acid and cardiovascular risk marker; hs-CRP: high-sensitivity C-reactive protein; NO: nitric oxide; MMP-9: matrix metalloproteinase-9; TSH: thyroid-stimulating hormone; T3: triiodothyronine; T4: thyroxine; C3: complement component 3; OPN: osteopontin; CD44: cell adhesion molecule and osteopontin receptor; OPN–CD44: osteopontin–CD44 complex; Granzyme B: proteolytic enzyme released by cytotoxic T lymphocytes (CTLs) and NK cells

Inflammatory cytokines were the most frequently evaluated markers (n=15), followed by immune system cells (n=6) adipokines (n=3), inflammatory proteins (n=3), oxidative stress markers (n=3), chemokines (n=2), metabolic-hormonal markers (n=2), autoantibodies (n=1), and others (n=5), such as thyroid markers and complement system components.^(10-28)^ The types and implications of these biomarkers in infertility among women with PCOS are detailed in chart 2.

The ELISA method was the most frequently used for measuring cytokines, adipokines, hormones, and other serum biomarkers.^(10-13,15-23,26-28)^ Flow cytometry was employed to analyze immune cell subpopulations, such as T lymphocytes, NK cells, and MAIT cells. Gene expression techniques (RT-qPCR) focused on investigating inflammatory pathways and inflammasome activation.^(10,13-15,19,28)^ Other methods included immunohistochemistry, spectrophotometry/colorimetry, and specific assays such as the Bio-Plex® multiplex bead-based immunoassay, FRET, and SRID.^(24,25,27,29)^ In addition, some studies mentioned the use of commercial kits (Aviscera Bioscience; Pishtaz Teb) for the analysis of adipokines and hormones.^(20)^

### Aspects of chronic inflammation and immunological dysfunction associated with reproductive function

Low-grade chronic inflammation was the most prevalent cause of infertility among women with PCOS. It was characterized by the persistent elevation of proinflammatory cytokines and immunoregulatory imbalance, with a direct impact on reproductive function (Chart 3).^(11-27)^ Based on the analysis of the included articles, chronic inflammation in reproductive organs was primarily described in the ovaries (n=7) and the endometrium (n = 5), with no specific reports involving the fallopian tubes, uterine body, or cervix. None of the studies addressed the role of macrophages in chronic inflammation associated with infertility in women with PCOS. Regarding NK cells, only two studies (n=2) investigated their involvement in this context.^(10-15,21-23,25,28)^ Additional information on the mentioned biological sites and their relationship with reproductive function is presented in chart 3.

**Chart 3:** Aspects of chronic inflammation and immune dysfunction in the reproductive function of women with PCOS

Biological site	Main findings	Cells involved	Impact on reproductive function	n(%)
Ovaries^(10,11-14,21,22)^	Inflammation in the follicular microenvironment and ovarian tissue	↑ IL-17 (FF); ↓ MAIT cells; Alterations in granulosa cells; ↑ IL-6, ↑ MCP-1, ↓ TNF-α	Impaired immunoregulation; chronic inflammatory state; impact on oocyte maturation and follicular development; ovarian dysfunction	7(36.8)
Endometrium^(21-23,25,28)^	↑ Inflammation in endometrial tissue	↑ expression of inflammatory mediators; ↓ uterine NK cells (late luteal phase)	↓ uterine receptivity and embryo implantation rate	5(26.3)

↑ increase; ↓ decrease; FF: Follicular fluid

### Hormonal aspects associated with the modulation of the immune response and their contribution to infertility

The analysis of the included studies identified six main categories related to hormonal and metabolic aspects that modulate the immune response and contribute to infertility in women with PCOS (Chart 4).

**Chart 4:** Hormonal and metabolic aspects associated with the modulation of immune response and infertility in women with PCOS

Category	Main findings	Impact on reproductive function	N(%)
Diabetes and/or insulin resistance^(11,13-17,20,21,24-27)^	Metabolic dysfunction associated with altered immunoregulation	Worsening of reproductive prognosis	12(63.2)
Hyperinsulinemia^(11,13,14,17,20,21,25,26)^	Associated with insulin resistance and alterations in the inflammatory cytokine profile	Immunological and metabolic alterations	8(42.1)
Hyperandrogenism^(11,13,14-17,18-21,23-26,28)^	↑ Testosterone and/or DHEAS; ↑ pro-inflammatory mediators	Ovarian microenvironment dysfunction	13(68.4)
Estrogen/Progesterone imbalance^(11,13-21,23-26,28)^	Alterations in sex hormones	↓ endometrial receptivity and ovulatory alterations	16(84.2)
Obesity^(11-17,20-27)^	Risk factor for systemic inflammation and hormonal alterations	Worsening of metabolic and hormonal dysfunctions	15(78.9)
Oxidative stress^(14,21,22,26,27)^	Alterations in redox balance	Impact on oocyte maturation and endometrial receptivity	5(26.3)

↑ increase; ↓ decrease

The category of diabetes and/or IR was addressed in twelve studies (n = 12), whereas hyperinsulinemia, often associated with IR, was reported in eight studies (n = 8).^(11,13-17,20,21,24-27)^

Hyperandrogenism, characterized by elevated levels of testosterone and/or Dehydroepiandrosterone Sulfate (DHEAS), was identified in thirteen studies (n=13), whereas imbalances in sex hormones, such as estrogen and progesterone, were reported in sixteen studies (n = 16), with repercussions for endometrial receptivity and ovulation.^(11,13-21,23-28)^

Obesity was discussed in fifteen studies (n=15), being identified as a risk factor for systemic inflammation and the worsening of hormonal alterations. In addition, oxidative stress was described in five studies (n = 5).^(11-17,20-27)^ The "other" category was not represented among the analyzed studies. The impact of these conditions on the reproductive function of women with PCOS is described in chart 4.

### Therapeutic strategies related to the immune system

Of the nineteen studies included, three addressed potential interventions or proposals related to the modulation of the immune response and the reproductive microenvironment in PCOS.^(21,25,26)^

## Discussion

The findings of this study demonstrate the multifactorial complexity of infertility in PCOS, highlighting the central role of immunological dysregulation associated with metabolic and hormonal imbalances. This complex feedback state is exacerbated by associated health conditions, such as IR/diabetes and obesity, which end up developing a chronic inflammatory process and an environment hostile to reproductive function.

Overall, a predominance of studies conducted in Asian countries is highlighted, especially in East Asia and the Middle East, and only two carried out in the United States. This distribution suggests greater scientific interest in the topic in regions with a high prevalence of reproductive challenges, whereas there is an underrepresentation of research originating from Western countries, such as the absence of publications from Brazil, constituting a gap to be explored in future investigations.

The literature points to inflammation as one of the pillars of the pathophysiological mechanisms responsible for reproductive dysfunction.^(11-27)^ Low-grade chronic inflammation establishes a vicious cycle of immunometabolic dysregulation, in which mediators such as TNF-α, secreted by macrophages and visceral adipose tissue, activate NF-κB signaling and stimulate excessive androgen production by theca cells.^(12,15,20,21,23)^

The resulting hyperandrogenism reduces sex hormone–binding globulin (SHBG), promotes abdominal fat accumulation, and perpetuates the inflammatory state. Consequently, in the ovary, these alterations contribute to abnormal follicular growth and inadequate oocyte maturation, while in the endometrium, inflammation, exacerbated by obesity, compromises uterine receptivity and decreases the likelihood of embryo implantation.^(21)^ These findings corroborate previous evidence indicating that persistent immune system activation driven by endocrine–metabolic imbalance plays a central role in PCOS-related infertility.^(2)^

In this context, significant alterations are observed in different cellular subpopulations and in molecules related to immunoregulation, such as increased levels of TNF-α, IL-6, IL-1β, IL-18, MCP-1, and PON-1, along with decreased levels of regulatory cytokines, including IL-27, IL-35, and IL-37.^(10,12,22)^ These findings demonstrate that the persistent activation of inflammatory pathways, combined with an imbalanced endometrial immune response and cytokine-, adipokine- (such as PGRN and CTRP6), and inflammatory protein (C3) mediated follicular dysfunctions, contributes to hormonal dysregulation and establishes an unfavorable environment for ovulation, fertilization, implantation, and pregnancy maintenance.^(20)^

The reduction of regulatory cytokines has also proven to be relevant in the onset of PCOS. Anti-inflammatory cytokines, such as IL-10, IL-27, IL-38, and IL-1Ra, were increased in some studies.^(10,26)^ This is possibly due to an attempt to contain the chronic inflammation present in the disease. On the other hand, anti-inflammatory cytokines such as TGF-β, NK cell-regulating cytokines (IL-15), and IL-22 were reported to be reduced in some women.^(12,26,28)^

IL-15, which is fundamental for the differentiation and function of uterine Natural Killer (uNK) cells, was shown to be decreased in the endometrium, suggesting impairment of the immunomodulation required for embryonic implantation.^(28)^ In contrast to an increase in NK cells in peripheral blood, reflecting systemic inflammation.^(15)^ This functional imbalance was shown to be associated with the presence of infertility, suggesting high peripheral cytotoxicity.^(15,28)^ Whereas IL-22, a protective cytokine that is important with regard to the development of IR, was found to be associated with ovarian dysfunction and anovulation.^(12)^ The final result of these mechanisms clearly demonstrates how PCOS and chronic inflammation are directly interconnected in reproductive outcomes.

When assessing the cytological context of the immune system, several studies have reported alterations in cells that play key roles in inflammation control. Some authors identified a reduction in regulatory cells such as Tregs, MAIT cells, CD4^+^ T cells, NK CD56^bright/CD16^–^ cells, Th2 lymphocytes, and DCs.^(10,13,19,28)^ Conversely, from another perspective, an increase in cells involved in immune system activation, such as CD3^+^ T cells, Th1, and Th17 lymphocytes, was observed.^(19)^ Thus, it is important to highlight that, in the follicular microenvironment and ovarian tissue, elevated levels of IL-17 were observed in the follicular fluid, associated with IR and obesity, implying impaired immunoregulation and promoting a chronic inflammatory state.^(13)^ These alterations demonstrate an imbalance in the cells responsible for orchestrating immune activity, representing yet another pillar sustaining the heightened inflammatory state in PCOS.

Still in this scenario, the paradoxical behavior of Natural Killer (NK) cells is highlighted, which showed a divergent pattern between systemic circulation and the reproductive microenvironment. An increase in NK cells was observed in peripheral blood, reflecting systemic inflammation, in contrast to a decrease in uNK in endometrial tissue. This functional imbalance was shown to be associated with the presence of anovulation, hyperandrogenism, and infertility, suggesting high peripheral cytotoxicity.^(15,28)^

IR plays a central role in this process and may be induced by systemic inflammation and oxidative stress, even in lean women with PCOS. IR enhances androgen secretion, impairs progesterone production, and exacerbates the autoimmune response, leading to ovulatory failure, compromised oocyte quality, and implantation difficulties.^(20,24,25,27)^ Consequently, characterized by an altered immunological status, these patients experience a state of persistent immune stress.^(15)^

Indeed, there is evidence that inflammation induced by IR and other associated health conditions directly affects the ovarian and endometrial microenvironment through the activation of pathways such as NF-κB and the NLRP3 inflammasome, impairing granulosa cell function and increasing oxidative stress.^(14,25)^ Thus, the interaction among chronic inflammation, IR, and endocrine dysfunctions constitutes a self-perpetuating mechanism that culminates in infertility at different levels.

According to Carvalho (2019),^(5)^ the main cause of reproductive dysfunction in this syndrome is normoestrogenic normogonadotropic anovulation. In light of the studies analyzed, it is understood that this anovulation results from a complex interaction between hormonal dysregulation and an exacerbated immune response, which reinforces the relevance of the altered immunometabolic environment as a cofactor in follicular alteration, impairment of embryo receptivity, and imbalance of the aforementioned immunoregulatory cytokines.^(13,26)^

The chronic anovulation observed in PCOS does not result solely from hyperandrogenism and altered gonadotropin secretion, but also from a dysregulated immunological microenvironment that compromises follicular dynamics. The reduction of regulatory cells, such as Tregs and CD56^bright NK cells, together with an increase in pro-inflammatory subpopulations, such as Th1 and Th17 cells, creates an imbalance between anti- and pro-inflammatory cytokines, impairing follicular rupture and ovulation.^(19,28)^ This scenario leads to granulosa cell dysfunction, decreased progesterone production, and failure in the proper formation of the corpus luteum, thereby perpetuating anovulatory cycles.^(24,25)^ Thus, the altered immunological status acts in the maintenance of chronic anovulation, potentiating the described endocrine and metabolic effects and directly compromising female fertility.^(20,21)^

Given the relevance of immunological processes in the pathophysiology of PCOS, their modulation represents a promising strategy for managing the syndrome. In this regard, studies involving murine models of PCOS have shown that metformin and gliclazide are capable of suppressing the expression of NF-κB, IL-6, and NrF2, demonstrating their modulatory effects on these inflammatory markers associated with the condition.^(10,25,29)^ Similarly, semaglutide may also reduce the expression of NF-κB, TNF-α, IL-6, and IL-1β, in addition to improving follicular cystic dilation and restoring reproductive cyclicity in murine models with induced PCOS.^(30)^

In parallel, the included studies presented methodological limitations that affect the generalization of the findings. In several studies, there were small sample sizes, studies conducted in single centers or within restricted geographical contexts, absence of a control group, or inadequacy of the control group.^(10,11,13-17,19-21,26-28)^

The predominance of cross-sectional designs limited causal inference between the evaluated markers and reproductive outcomes, as the frequent absence of longitudinal follow-up precluded assessment of the direct clinical impact of these alterations on fertility.^(11,12,18,20,21,23,25-27)^ There was relevant methodological heterogeneity, such as variation in the menstrual cycle phase during sample collection and lack of standardization in the controlled hormonal variables.^(19,24,25,28)^ In addition, the absence of multivariate analysis to control for confounding factors and the single measurement of hormonal and inflammatory parameters were identified as additional weaknesses that may have influenced the conclusions.^(17,18)^

The absence of a direct approach addressing therapeutic strategies specifically aimed at immunological factors related to infertility in women with PCOS was observed, which limits the clinical applicability of the findings.^(10-13,15-18,20,22-24,27,28)^ Shruthi et al. (2024)^(26)^ suggested the neutralization of IL-27 and IL-38 as a future immunomodulatory approach; Shirvanizadeh et al. (2023)^(21)^ recommended lifestyle modifications (diet, exercise, and weight control) and possible pharmacotherapy to reduce inflammation; and Koc et al. (2017)^(25)^ mentioned metformin as a potential NF-κB modulator. However, in all these cases, there was no practical application or evaluation of outcomes, which restricts the clinical applicability of the findings.

Finally, regarding the limitations inherent to this scoping review, the risk of publication bias is highlighted, characterized both by the overrepresentation of studies reporting positive associations between immunological/inflammatory markers and infertility in women with PCOS and by a geographic bias. This regional concentration may reflect local research priorities, specific population characteristics, and distinct healthcare contexts, which limits the generalizability of the findings to other populations.

To mitigate these gaps, broad inclusion criteria and a comprehensive search strategy across multiple databases were adopted, as recommended by the JBI for Scoping Reviews, with the aim of reducing the risk of excluding relevant evidence. Additionally, the detailed qualitative synthesis and the grouping of markers by biological categories allowed for a consistent and contextualized interpretative analysis, compensating for the impossibility of a quantitative synthesis in view of the marked methodological diversity of the primary studies.

In general, this review highlights the scarcity of recent studies in the Brazilian context and reinforces the need for integrated approaches that concomitantly consider the immunological, metabolic, and hormonal axes. From a future perspective, these findings provide a solid conceptual basis for the development of new research in the field of reproductive immunology and contribute to the qualification of care in addressing the health needs of this group.

## Conclusion

The infertility in women with PCOS results from a complex interplay between immune dysregulation and endocrine–metabolic disturbances. The findings demonstrate that low-grade chronic inflammation, characterized by increased levels of pro-inflammatory cytokines and an imbalance in immunoregulatory responses, compromises both the follicular microenvironment and endometrial receptivity and quality. These mechanisms, associated with IR, hyperandrogenism, obesity, and oxidative stress, establish a feedback loop that perpetuates reproductive dysfunction.

## Data Availability

The research data are described in the article presented.
