# Supplementary material for: Interconnections between the immune system and infertility in women with polycystic ovary syndrome: a scoping review
Source: Rev Bras Ginecol Obstet. 2026 Oct 20;48:e-rbgO39. doi: 10.61622/rbgo/2026rbgo39 (PMC13690368; doi:10.61622/rbgo/2026rbgo39)
Supplement: Supplementary file 1 [file 1806-9339-rbgo-48-e-rbgo39-Suppl01.pdf]

Supplementary material

Chart 1S. Search strategies and results obtained by database

Database	Search strategy	Results 11.01.2025
Pubmed	((“Polycystic Ovary Syndrome”) AND (english[Filter] OR portuguese[Filter] OR spanish[Filter])) AND ((“Infertility” OR “Anovulation” OR “Amenorrhea”) AND (english[Filter] OR portuguese[Filter] OR spanish[Filter])) AND ((“Immune System” OR “Immune System Phenomena” OR “Inflammation” OR “cytokines”) AND (english[Filter] OR portuguese[Filter] OR spanish[Filter]))<br><br>Filters: English, Portuguese, Spanish, from 2010 - 2024	344
Embase	(‘polycystic ovary syndrome’) AND (‘infertility’ OR ‘anovulation’ OR ‘amenorrhea’) AND (‘immune system’ OR ‘immune system phenomena’ OR ‘inflammation’ OR ‘cytokines’) AND [embase]/lim AND [2010-2024]/py AND ([english]/lim OR [portuguese]/lim OR [spanish]/lim)	574
Virtual Health Library (VHL)	(“Polycystic Ovary Syndrome”) AND ((infertility OR anovulation OR amenorrhea)) AND ((“Immune System” OR “Immune System Phenomena” OR inflammation OR cytokines)) AND db:(“MEDLINE” OR “BINACIS” OR “MedCarib”) AND (year_cluster:[2010 TO 2024]) AND instance:”lilacsplus”	310
Lilacs	(“Polycystic Ovary Syndrome”) AND ((infertility OR anovulation OR amenorrhea)) AND ((“Immune System” OR “Immune System Phenomena” OR inflammation OR cytokines)) AND db:(“LILACS”) AND (year_cluster: [2010 TO 2024]) AND instance:”lilacsplus”	02
Web of Science	((ALL=(“Polycystic Ovary Syndrome”)) AND ALL=(“Infertility” OR “Anovulation” OR “Amenorrhea”)) AND ALL=(“Immune System” OR “Immune System Phenomena” OR “Inflammation” OR “cytokines”)	378
Scopus	(TITLE-ABS-KEY (“Polycystic Ovary Syndrome”) AND TITLE-ABS-KEY ((infertility OR anovulation OR amenorrhea)) AND TITLE-ABS-KEY ((“Immune System” OR “Immune System Phenomena” OR inflammation OR cytokines))) AND PUBYEAR > 2009 AND PUBYEAR < 2025 AND (LIMIT-TO (LANGUAGE , “English”) OR LIMIT-TO (LANGUAGE , “Spanish”))	520
